# PI3K/Akt-independent negative regulation of JNK signaling by MKP-7 after cerebral ischemia in rat hippocampus

**DOI:** 10.1186/1471-2202-14-1

**Published:** 2013-01-02

**Authors:** JianXi Zhu, Wei Shen, Li Gao, Hao Gu, ShuTong Shen, Yi Wang, HuiWen Wu, Jun Guo

**Affiliations:** 1 Key Laboratory of Human Functional Genomics of Jiangsu Province, Nanjing Medical University, Nanjing, 210029, People’s Republic of China; 2 Department of Biochemistry and Molecular Biology, Nanjing Medical University, Nanjing, 210029, People’s Republic of China; 3 Department of Neurology, Brain Hospital Affiliated to Nanjing Medical University, Nanjing, 210029, China; 4 Laboratory Center for Basic Medical Sciences, Nanjing medical university Nanjing, Nanjing, 210029, People’s Republic of China

**Keywords:** Cerebral ischemia, JNK, PI3K/Akt, MKP-7

## Abstract

**Background:**

The inactivation of c-Jun N-terminal kinase (JNK) is associated with anti-apoptotic and anti-inflammatory effects in cerebral ischemia, which can be induced by an imbalance between upstream phosphatases and kinases.

**Result:**

Mitogen-activated protein kinase phosphatase 7 (MKP-7) was upregulated significantly at 4 h of reperfusion postischemia in rat hippocampi. By administration of cycloheximide or siRNA against mitogen-activated protein kinase phosphatase 7 (MKP-7) in a rat model of ischemia/reperfusion, an obvious enhancement of JNK activity was observed in 4 h of reperfusion following ischemia, suggesting MKP-7 was involved in JNK inactivation after ischemia. The subcellular localization of MKP-7 altered after ischemia, and the inhibition of MKP-7 nuclear export by Leptomycin B up-regulated JNK activity. Although PI3K/Akt inhibition could block downregulation of JNK activity through SEK1 and MKK-7 activation, PI3K/Akt activity was not associated with the regulation of JNK by MKP-7.

**Conclusions:**

MKP-7, independently of PI3K/Akt pathway, played a key role in downregulation of JNK activity after ischemia in the rat hippocampus, and the export of MKP-7 from the nucleus was involved in downregulation of cytoplasmic JNK activity in response to ischemic stimuli.

## Background

Ischemia/reperfusion brain damage is a major risk factor of a variety of serious human neurological disorders such as learning disabilities, cerebral palsy, epilepsy and seizures or even death [1]. c-Jun N-terminal kinase (JNK) is a potent mediator of inflammation and apoptosis [2]. After ischemia, the JNK signaling pathway is highly activated, promoting the transcription of ischemia related genes and ultimately resulting in the lesion and dysfunction of neurons [3,4]. Blockade of the JNK signaling pathway has been shown to protect neurons from ischemia/reperfusion injury and promote neuronal survival [5,6]. However, rapid inactivation of JNK following its activation by cerebral ischemia has also been observed [7]. Therefore, studying JNK inactivation may reveal the mechanism underlying the regulation of JNK activity after ischemia and support a new approach for treating ischemia/reperfusion injury.

The JNK signaling cascade is regulated by the balance between upstream kinases and phosphatases [8]. The JNK pathway is a mitogen-activated protein kinase (MAPK) cascade in which mixed lineage kinases (MLKs/MAPKKK) activate MAP kinase kinase 4/7 (MKK4/7), which then activate JNK [9]. Inhibiting MLK3 and MKK4/7 to block upstream kinase cascades involves JNK inactivation. Akt, known as a neuroprotective protein, can downregulate JNK activity through blocking an upsteam kinase [10]. Akt inhibits the ASK1–SEK1–JNK2 signal transduction pathway by inactivating SEK1 through Ser80 phosphorylation in response to glucose deprivation [11]. It has also been reported that the PI3K/Akt cascade negatively regulates the JNK pathway in PC12 cells by inhibiting MLK3 to downregulate MKK-7 [12]. Meanwhile, the activity of Akt is upregulated by ischemic pre-treatment and estrogen, which is commonly considered to have a protective role in ischemia induced injury [13,14].

Phosphatases also regulate JNK activity by direct dephosphorylation at residues Thr185 and Tyr183 [15]. Members of the MKP family, which includes ten proteins, dephosphorylate MAPKs at both phosphothreonine and phosphotyrosine residues simultaneously within the MAPK TXY (Thr-Xaa-Tyr) activation motif and share similar structural folding at the catalytic site, as well as contain critical kinase-interacting motifs (KIMs) conferring specific MAPK substrate specificity [16]. Individual MKPs generally show a substrate preference for one or more of the MAPKs. Among the MKPs, MKP-7 has a higher substrate specificity for JNK than for extracellular signal-regulated protein kinase (ERK) and P38 [17].

Some studies have demonstrated that the RNA interference (RNAi)-mediated ablation of MKP-7 increases the extent and duration of JNK activation induced by H_2_O_2_ stimulation in 293 T cells [18,19], while overexpression of MKP-7 in COS-7 cells blocks the activation of JNK in a dose-dependent manner [20]. Just as MAPKs are regulated by MKPs, in turn MKPs are also regulated by multiple factors. MKP-7 possesses a long C-terminal stretch containing both a nuclear export signal (NES) and a nuclear localization signal (NLS), which mediate their subcellular localization and nuclear-cytoplasmic shuttling by transport proteins [20]. Acute oxidative stress has been reported to lead to redistribution of MKP-7 from the nucleus into the cytoplasm and a reduction in cytoplasmic p-JNK [21].

In this study, the relationships between upstream kinases and phosphatases were explored to determine the key regulator in JNK inactivation following cerebral ischemia. The findings of this study suggest that MKP-7 plays a key role in JNK inactivation during and after ischemia, and this regulation occurs independently of PI3K/Akt pathway.

## Results

### Dual-phase phosphorylation of JNK induced by cerebral ischemia coincides with Akt-induced SEK1 and MKK-7 phosphorylation in the rat hippocampus

**Figure 1:**
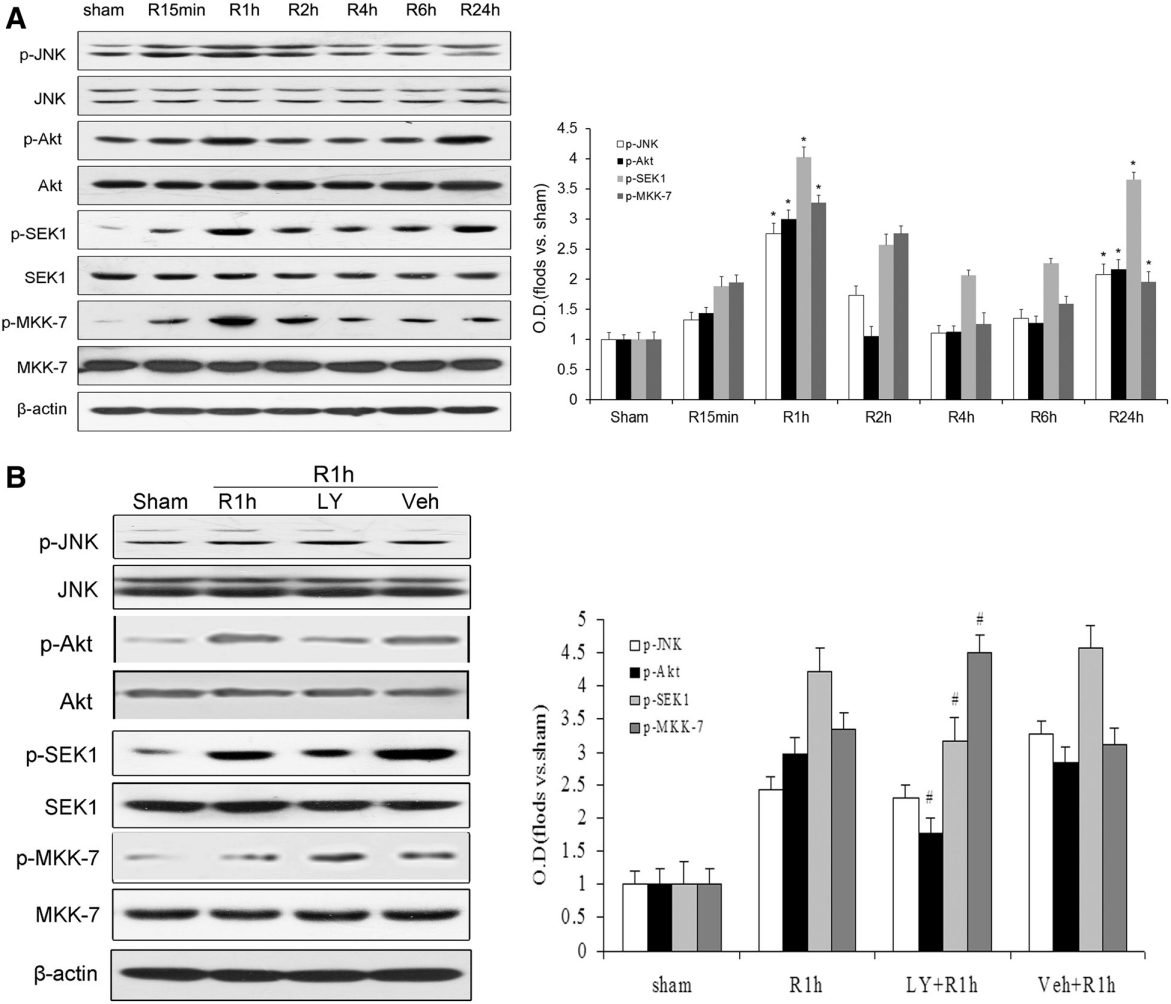
**Western blot analysis of JNK, Akt, SEK1 and MKK-7 activities in response to ischemia at different times after reperfusion and effect of PI3K/Akt inhibitor LY294002 after 1 h of reperfusion.** Proteins were extracted and measured using antibodies against p-JNK (Thr183/Tyr185), p-Akt (Ser473), p-MKK-7 (Ser271/Thr275), p-SEK1 (Ser80), JNK, Akt, MKK-7 and SEK1. **A**: Phosphorylation state of JNK, Akt, MKK-7 and SEK1 after different reperfusion times (15 min, 1, 2, 4, 6 and 24 h) following 10 min of ischemia. **B**: Determination of p-JNK, p-MKK-7 and p-SEK1 levels in animals administered LY294002 (LY) compared with sham and control groups (vehicle, Veh). ODs are presented as means ± SEM (n = 4) and expressed as magnitudes of change versus sham control or control. *P < 0.05. versus sham control; #*P* < 0.05, comparison between the 1-h chemically treated reperfusion groups and their respective vehicle controls.

It is well known that cerebral ischemia highly induces activation of the JNK pathway through phosphorylation, leading to apoptosis and dysfunction of neurons. However, JNK activity has been observed to obviously decrease following its activation by cerebral ischemia [7], but the precise mechanism remains unclear. To determine the underlying cause of the abrupt JNK inactivation following cerebral ischemia, phosphorylation of JNK at Thr183/Tyr185 and its upstream molecules Akt at Ser473, SEK1 at Ser80 and MKK-7 at Ser 271/Thr275 were examined in the rat hippocampus following ischemia. Rats were subjected to four-vessel occlusion, with 10 min of ischemia followed by reperfusion for 15 min, 1, 2, 4, 6 and 24 h. As shown in Figure 1A, phosphorylation of JNK showed a biphasic pattern of elevation during reperfusion, gradually increasing from 15 min (*P* < 0.05), peaking at 1 h (*P* < 0.05), but then decreasing significantly at 4 h (*P* < 0.05) before elevating again at 24 h (*P* < 0.05). Meanwhile, the phosphorylation of Akt, SEK1 and MKK-7 peaked at 1 h (*P* < 0.05), showing that phosphorylation of these proteins may be associated with JNK. After peaking at 1 h, their phosphorylation sharply decreased at 4 h (*P* < 0.05) and was elevated again after 24 h (*P* < 0.05). Meanwhile, no changes were observed in the levels of total JNK, Akt, SEK1, MKK-7 and β-actin in each group during the reperfusion (*P* > 0.05, Figure 1). These results demonstrated that cerebral ischemia induced the dual-phase phosphorylation of JNK, and the phosphorylation patterns of Akt, SEK1 and MKK-7 showed the same trends as that of JNK after ischemia.

### PI3K/Akt inhibitor LY294002 increases SEK1 and MKK-7 activities but does not result in upregulation of JNK activity following cerebral ischemia

The results above showed that Akt phosphorylation was associated with the JNK signaling cascade. However, to determine the exact role played by Akt in the JNK cascade in cerebral ischemia, the inhibitor LY294002 (LY) was employed to block the PI3K/Akt pathway. Rats underwent four-vessel occlusion and endured a 10-min period of ischemia, followed by reperfusion for 1 h. Phospho-specific antibodies were employed, and changes in JNK, Akt, SEK1 and MKK-7 activities were examined in the hippocampus of rats after the administration of LY or vehicle. The inactivated form of SEK1 is phosphorylated at Ser80, while the activated form of MKK7 is phosphorylated at Ser 271/Thr275. As shown in Figure 1B, in the 1-h reperfusion groups, a significant decline of p-Akt (Ser473) and p-SEK1 (Ser80) was observed in those rats treated with the LY compared with the vehicle-treated rats (*P* < 0.05). Meanwhile, the level of p-MKK-7 (Ser271/Thr275) was significantly elevated in the LY group compared with the vehicle group. However, the levels of activated JNK differed little between the 1-h reperfusion groups with and without the administration of LY. This result suggests that the PI3K/Akt inhibitor LY could downregulate Akt activity following cerebral ischemia, which increased the activity of SEK1 and MKK-7 but did not lead to the elevation JNK activity. Therefore, we hypothesized that another mechanism must be involved in JNK inactivation at 1 h reperfusion postischemia.

### MAPK phosphatase is involved in JNK inactivation following cerebral ischemia

**Figure 2:**
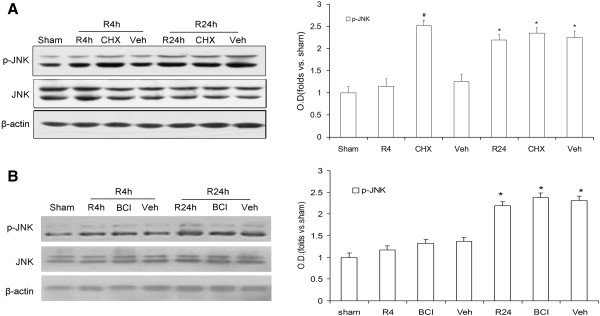
**Effect of CHX or BCI on p-JNK in rat hippocampi following ischemia/reperfusion.** Proteins were extracted and measured using antibodies against p-JNK (Thr183/Tyr185) and JNK. **A**: Effect of CHX on p-JNK (Thr183/Tyr185) during 4 h or 24 h of reperfusion following 10 min of ischemia. **B**: Effect of BCI on p-JNK after 4 h and 24 h of reperfusion following ischemia. OD data are presented as means ± SEM (n = 4) and expressed as magnitudes of the alteration versus sham control or control. *P < 0.05. versus sham control; ^#^*P* < 0.05, comparison between the chemically treated 4-h and 24-h reperfusion groups and their vehicle control.

Activated JNK promotes ischemic injury related protein expression through phosphorylation of transcription factors such as c-jun. In order to control appropriate gene transcription, the activity of JNK must be tightly regulated by the actions coordinated between protein kinases and phosphatases. MKPs comprise a subset of protein tyrosine phosphatases, which can dephosphorylate both phosphothreonine and phosphotyrosine residues. Cycloheximide (CHX) is usually considered a protein synthesis inhibitor, and some studies have also shown that it can inhibit the activity of MKPs [22]. To explore whether MKPs were involved in JNK inactivation after ischemia in the rats, CHX was employed in this study. As shown in Figure 2A, levels of JNK activity were significantly elevated in the CHX group compared with the vehicle group at 4 h after reperfusion (*P* < 0.05). BCI, an allosteric inhibitor of Dusp6 [23], was also used in this study, however, the levels of JNK activity were not changed in the BCI group compared with the vehicle group at 4 h after reperfusion (Figure 2B, *P* > 0.05). Furthermore, activated JNK levels differed little between the 24-h reperfusion groups with and without the administration of MKPs inhibitor (CHX or BCI), with both groups exhibiting high levels (*P* > 0.05). This result suggested that a few MKPs but not Dusp6 were involved in JNK inactivation at 4 h reperfusion after ischemia.

### MKP-7 participates in JNK inactivation in the rat hippocampus after cerebral ischemia

**Figure 3:**
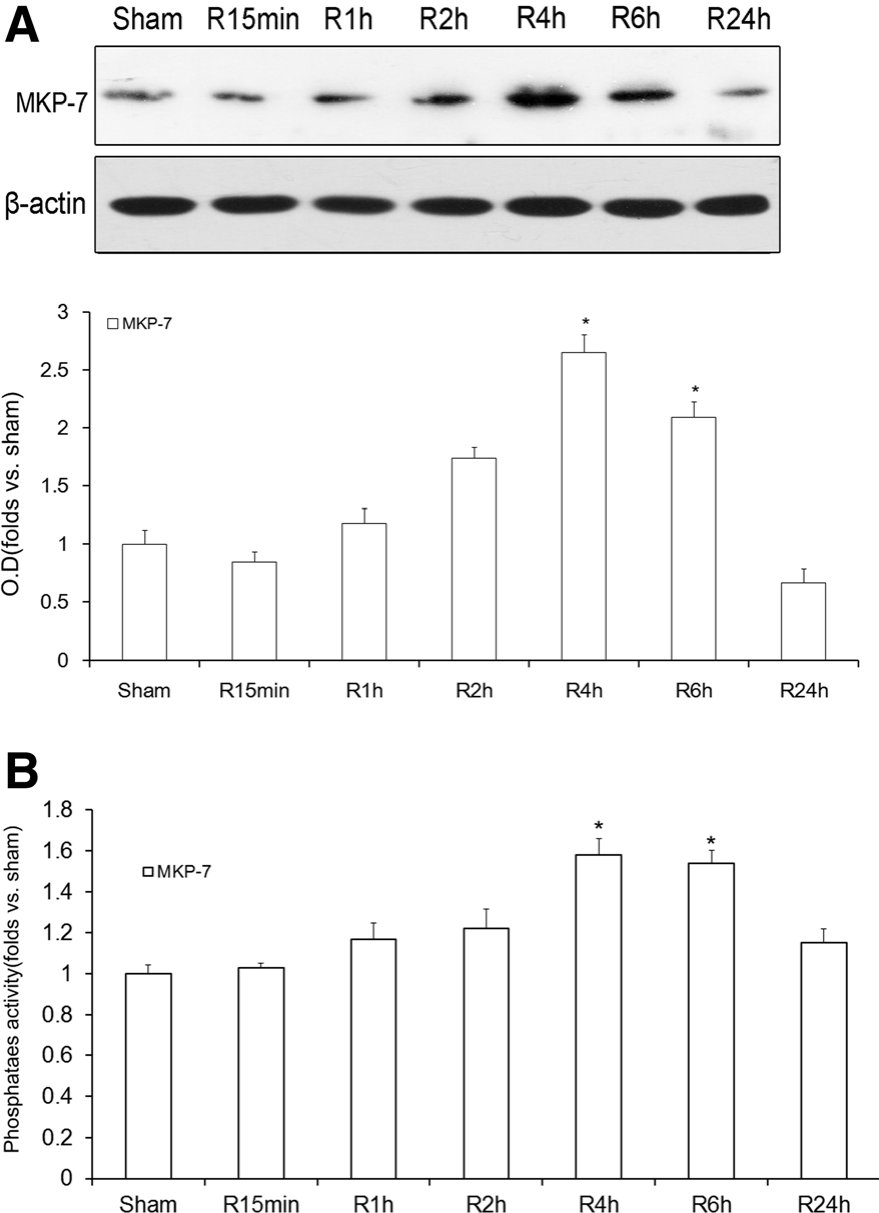
**Temporal curve of MKP-7 protein levels and activities during different reperfusion times following cerebral ischemia.** Proteins were extracted and measured using antibodies against MKP-7. MKP-7 was purified by immunoprecipitation, and its activity was measured using an alkaline phosphatase assay. **A**: Time curve of MKP-7 levels during different reperfusion times (15 min, 1, 2, 4, 6 and 24 h) following 10 min of ischemia. **B**: Temporal curve of MKP-7 activity after different reperfusion times (15 min, 1, 2, 4, 6 and 24 h) following 10 min of ischemia. OD data are presented as means ± SEM (n = 4) and expressed as magnitudes of the alteration versus sham control or control [*P < 0.05 versus sham control].

We wanted to further identify which member of the MKP family was involved in the downregulation of JNK activity at 4 h after reperfusion. MKP-7 is a JNK-specific phosphatase. A previous study showed that the level of JNK phosphorylation is significantly prolonged when the expression of endogenous MKP-7 is ablated by siRNA [18,19]. In order to explore the role of MKP-7 in JNK inactivation after ischemia, its cytoplasmic level and activity were observed after ischemia/reperfusion. As shown in Figure 3A, the levels of cytoplasmic MKP-7 reached peak levels at 4 h (*P* < 0.05). However, it decreased obviously at 24 h again (*P* < 0.05). This result showed that the trend of cytoplasmic MKP-7 protein level followed the opposite pattern as that of JNK activity after ischemia. As shown in Figure 3B, MKP-7 activity after ischemia was also observed to slightly increase from 15 min to 2 h (*P* < 0.05) and reached a peak level at 4 h (*P* < 0.05). However, it decreased significantly at 24 h (P < 0.05). The trend in MKP-7 activity was similar to that of the corresponding cytoplasmic protein level and again displayed the opposite pattern to that of JNK activity.

**Figure 4:**
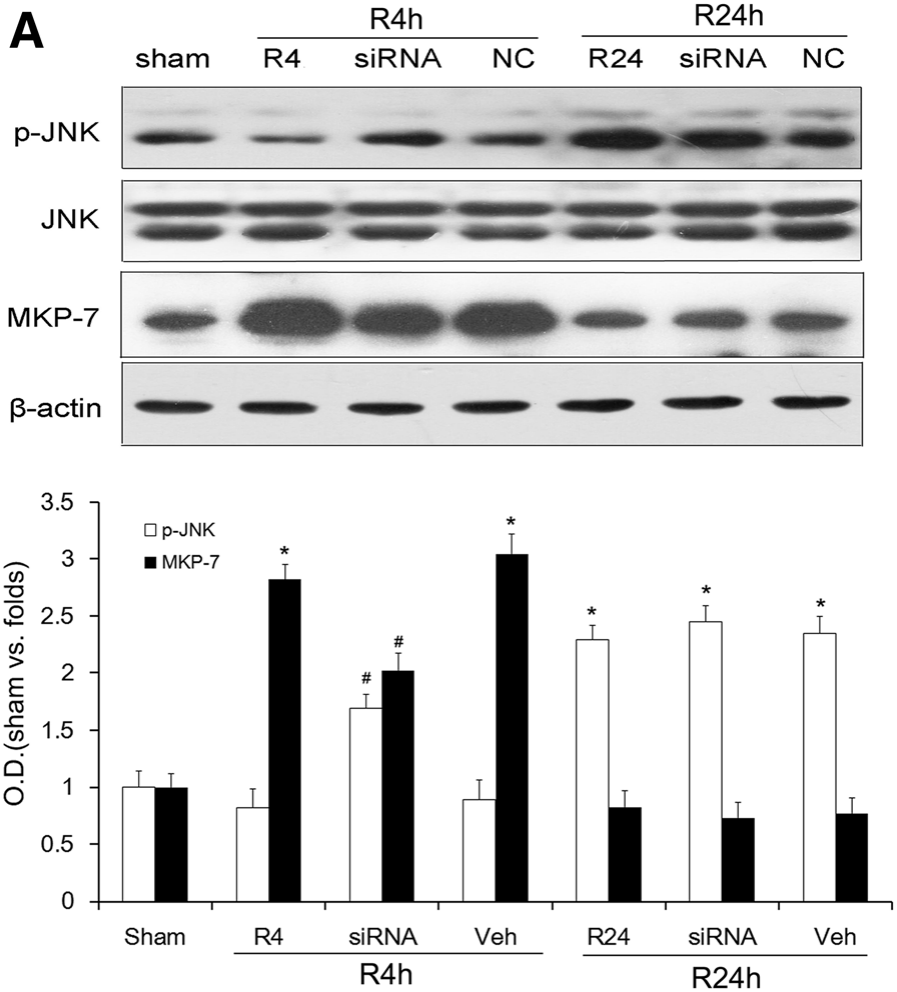
**Effect of administration MKP-7 siRNA on the phosphorylation state of JNK (Thr183/Tyr185) after 4 or 24 h of reperfusion post-ischemia.** Non-coding (NC) siRNA was used as a control. Proteins were extracted and measured using antibodies against p-JNK (Thr183/Tyr185), JNK and MKP-7 **A**: Determination of p-JNK (Thr183/Tyr185) and MKP-7 levels with and without MKP-7 siRNA after 1 or 4 h of reperfusion following 10 min of ischemia. OD data are presented as means ± SEM (n = 4) and expressed as magnitudes of the alteration versus sham control or control. *P < 0.05, versus sham control; #*P* < 0.05, comparison between chemically treated 4-h and 24-h reperfusion groups and their vehicle control.

The results presented above indicated that MKP-7 could participate in the inhibition of JNK activity after ischemia. To further clarify the role of MKP-7 in down-regulation of JNK activity after ischemia, MKP-7 siRNA was employed to decrease the amount of cytoplasmic MKP-7. As shown in Figure 4, JNK activity was markedly reduced in the 4-h reperfusion control and 4-h reperfusion vehicle groups compared with that in the 1-h reperfusion control. As expected, this inhibitory effect was sufficiently relieved after 4 h of reperfusion regardless of MKP-7 siRNA treatment. However, in contrast with the changes in p-JNK, this phosphorylation state was maintained after 24 h of reperfusion, and administration of MKP-7 siRNA was unable to reverse it (*P* > 0.05). This result confirmed our hypothesis that MKP-7 was involved in JNK inactivation during the 4 h but not 24 h of reperfusion post-ischemia.

### Ischemia/reperfusion induces MKP-7 nuclear export to downregulate JNK activity

**Figure 5:**
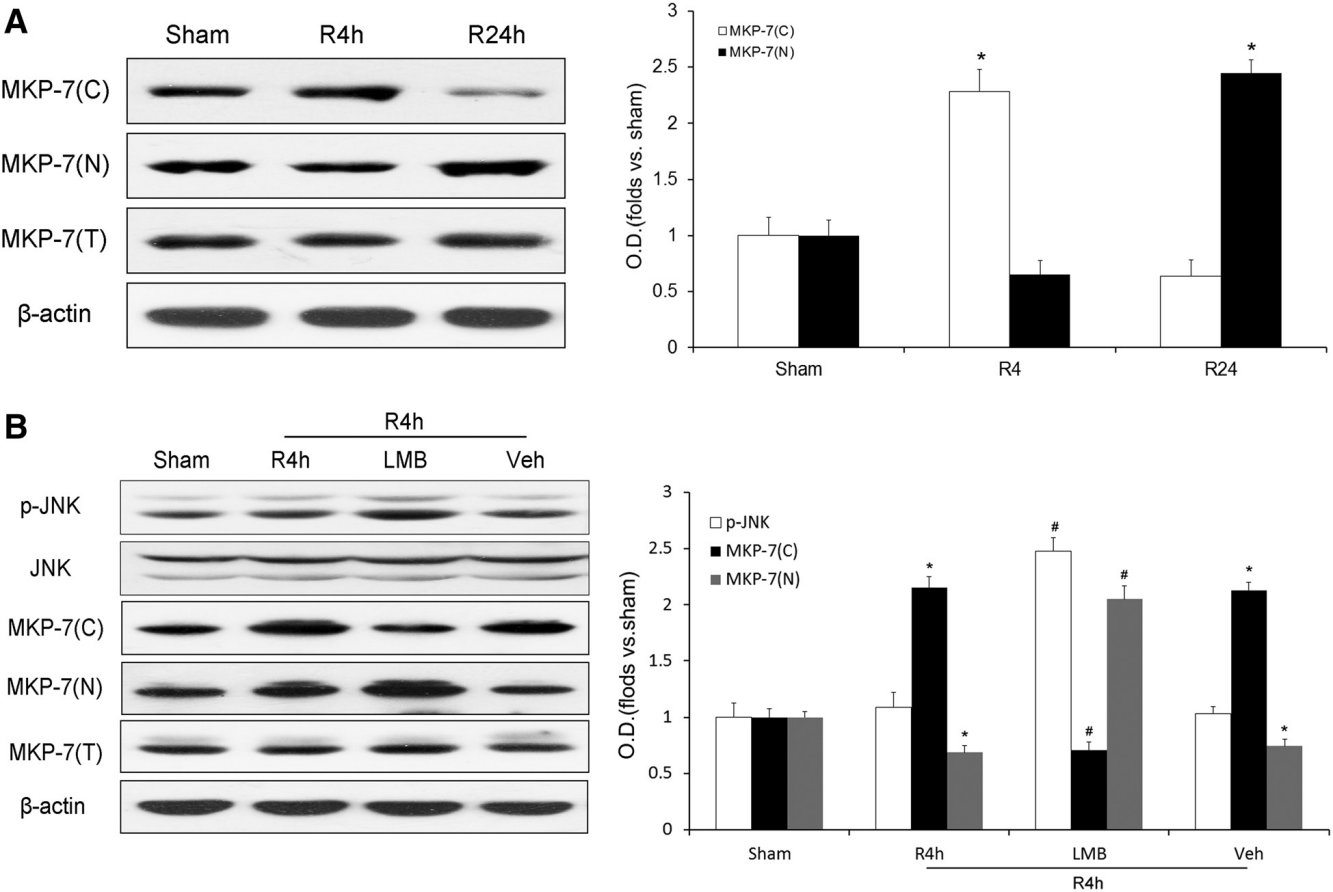
**Western blot analysis of the localization of MKP-7 in cytoplasm and nucleus after ischemia and the effect of inhibition of this MKP-7 translocation by LMB on JNK activity.** LMB administered in rat hippocampus as described in the Methods, and its vehicle (Veh) was used as a control. Proteins were obtained from the cytoplasm and nucleus and measured using antibodies against p-JNK (Thr183/Tyr185) and MKP-7. **A**: MKP-7 levels in cytoplasm and nucleus after 4 or 24 h of reperfusion following 10 min of ischemia. **B**: Effect of LMB on JNK activity in cytoplasm and levels of MKP-7 in cytoplasm and nucleus after 4 h of reperfusion post-ischemia. OD data are presented as means ± SEM (n = 4) and expressed as magnitudes of the alteration versus sham control or control. *P < 0.05, versus sham control; ^#^*P* < 0.05, comparison between chemically treated 4-h reperfusion groups and their vehicle control.

The results above showed that ischemia/reperfusion elevated the levels and activities of cytoplasmic MKP-7 to downregulate JNK activity at 4 h of reperfusion after ischemia. The next step was to investigate the potential mechanism of MKP-7 upregulation after ischemia. MKP-7 contains an intrinsic NES motif which mediates subcellular localization, and allows this protein to function as a nuclear-cytoplasmic shuttle protein upon stimulation by extracellular signals. To explore whether MKP-7 subcellular localization was altered during ischemia/reperfusion, the cytoplasmic and nuclear levels of MKP-7 were examined by western blot. As shown in Figure 5A, there was a striking increase in cytoplasmic MKP-7 and a decrease in nuclear MKP-7 in the 4-h reperfusion group compared with the sham group. However, cytoplasmic MKP-7 obviously decreased, while nuclear MKP-7 was strikingly elevated after 24 h of reperfusion. The results above suggested that MKP-7 was transported from the nucleus to cytoplasm at 4 h of reperfusion and was imported back to the nucleus at 24 h of reperfusion after ischemia.

As it was demonstrated above that nuclear export of MKP-7 increased its cytoplasmic levels at 4 h of reperfusion after ischemia, we further examined whether this export of MKP-7 was involved in JNK inactivation. LMB, a specific inhibitor of nuclear export that blocks binding between the NES and export receptor, causes nuclear accumulation of MKP-7 [21]. In this study, LMB was employed to identify the relationship between MKP-7 export and down-regulation of JNK activity. As shown in Figure 5B, there was a striking decrease in cytoplasmic MKP-7 and an increase in nuclear MKP-7 in the LMB group after 4 h of reperfusion. Meanwhile, as expected, the levels of JNK activity increased in the LMB group compared with the vehicle group. These results suggested that the export of MKP-7 was involved in JNK inactivation after 4 h of reperfusion after ischemia.

### Ischemia-induced activation of MKP-7 is independent of the PI3K/Akt pathway

**Figure 6:**
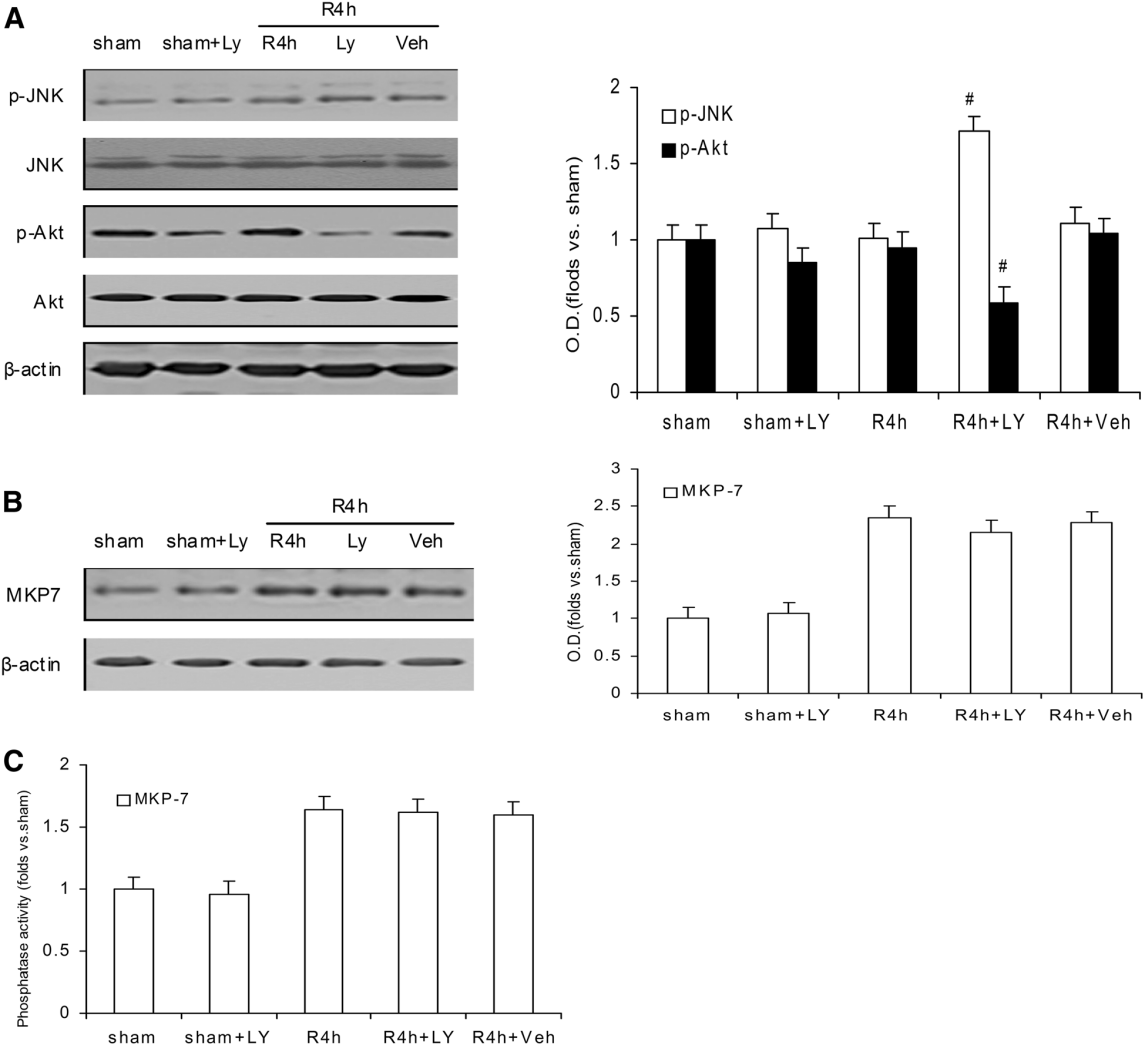
**Determination of JNK and Akt activities with and without LY294002 (LY) after 4 h of reperfusion after ischemia and effect of LY on the level and activity of MKP-7 after 4 h of reperfusion.A**. Effect of LY on Akt and JNK activities after 4 h of reperfusion. **B**. Effect of LY treatment on MKP-7 level after 4 h of reperfusion. **C**. Effect of LY treatment on MKP-7 activity after 4 h of reperfusion. ODs are presented as means ± SEMs (n = 4) and expressed as magnitudes of change versus sham control. *P < 0.05, versus sham control, # *P* < 0.05, comparison between chemically treated 4-h reperfusion groups and their vehicle control.

We also found that treatment with the PI3K/Akt inhibitor LY294002 induced a significant elevation in JNK activity during the 4 h of reperfusion (Figure 6A). Although there is much evidence to support the contention that JNK dephosphorylation/inactivation by MKP-7 is independent of PI3K/Akt pathway, some studies have suggested that PI3K/Akt cascade plays a key role in the downregulation of JNK activity [12,24]. Furthermore, whether PI3K/Akt pathway downregulates JNK via MKP-7 was previously unknown. In order to identify the relationship between inactivation of JNK by MKPs and the PI3K/Akt signaling pathway, the level and activity of MKP-7 were examined by administration of the PI3K/Akt inhibitor LY294002 (LY). As shown in Figure 6B, there was no difference between the LY and vehicle groups in terms of MKP-7 activity during the 4-h reperfusion. Meanwhile, the level of MKP-7 also differed little between the 4-h reperfusion groups with and without the administration of LY, both exhibiting high levels (Figure 6C). Furthermore, the protein level and activity of JNK and MKP-7 kept stable between the sham groups with and without the administration of LY. These results suggested that the MKP-7 downregulation of JNK activity after ischemia did not depend on the PI3K/Akt pathway.

## Discussion

Nerve cells involved in ischemia/reperfusion injury activate a complex system of signal transductions, in which activation of JNK plays an important role in cerebral lesion. Inactivation of JNK blocks phosphorylation of a wide range of transcription factors and decreases the expression of ischemia related proteins [25]. Therefore, the therapeutic potential of JNK inhibition is being investigated as an approach to protect cells from neurodegeneration and dysfunction [26].

Akt is an important cellular survival protein that protects cells from damage by inactivating JNK [27]. During ischemia/reperfusion, ischemic pre-conditioning and estrogen may activate Akt to protect neurons by downregulating JNK activity. Therefore, Akt is considered an important negative regulator of JNK activity [28]. However, in the current study, the evolution of Akt activity at 1 h of reperfusion did not result in JNK inactivation after ischemia and downregulation of Akt activity by PI3K/Akt inhibitor also did not induce increment of JNK activity at 1 h reperfusion. On the other hand, increases in the activity and level of MKP-7 at 4 h of reperfusion were observed and associated with JNK inactivation. Therefore, we propose that MKP-7 is an important newly defined regulator in the downregulation of JNK activity after ischemia.

The MKP-7 involves in the downregulation of JNK activity after ischemia in a PI3K/Akt independent manner. So it could be supposed that there are some differences between MKP-7 and Akt in their protective roles through JNK inactivation after ischemia. The increase of Akt activity to block the JNK cascade after ischemia is induced by external stimuli such as estrogen, anesthetic pre-conditioning and ischemic pre-conditioning [13,29,30]. However, the elevations in MKP-7 level and activity to inactivate JNK are induced by ischemia/reperfusion and not by other protective factors [31]. In contrast to the protection mechanism of Akt, the inhibition of JNK by MKP-7 provides a negative feedback regulatory mechanism which prevents excessive ischemia/reperfusion injury after ischemia. Essentially, ischemia/reperfusion induces a series of alterations in physiological conditions and activates the JNK signal cascade. Meanwhile, it also elevates the level and activity of MKP-7, which cause a feedback signal to inhibit JNK activity by direct dephosphorylation. While blockade of the JNK cascade induced by Akt is long-lasting and can alleviate ischemia/reperfusion injury [24], the administration of siRNA to specifically target MKP-7 showed no effect on JNK activity after 24 h of reperfusion. These results suggested that the inactivation of JNK by MKP-7 did not last throughout the 24 h after reperfusion. Therefore, the protective effect of MKP-7 may have been short-lived and could not substantially alleviate ischemia/reperfusion injury following cerebral ischemia.

The regulation of phosphatases can usually be observed as a change in protein levels, phosphatase activity and stabilization. Elevation of MKP-7 mRNA by serum has been shown to result in the increase of the MKP-7 protein level [32]. However, in this study, we found other regulatory mechanisms which elevated MKP levels. MKP-7 is a shuttle protein, and the exclusion of JNK-specific MKP-7 from the nucleus and its accumulation in the cytoplasm showed that it was exported from the nucleus after ischemia in the rat hippocampus. Since MKP-7 is a specific phosphatase of JNK, the change of its subcellular localization could have altered the level of p-JNK. Therefore, it is reasonable to conclude that the elevation of cytoplasmic MKP-7 after export from the nucleus ultimately led to the downregulation of JNK activity. This novel observation suggested that the altered subcellular localization of MKPs also led to the elevation of cytoplasmic MKP levels.

## Conclusions

**Figure 7:**
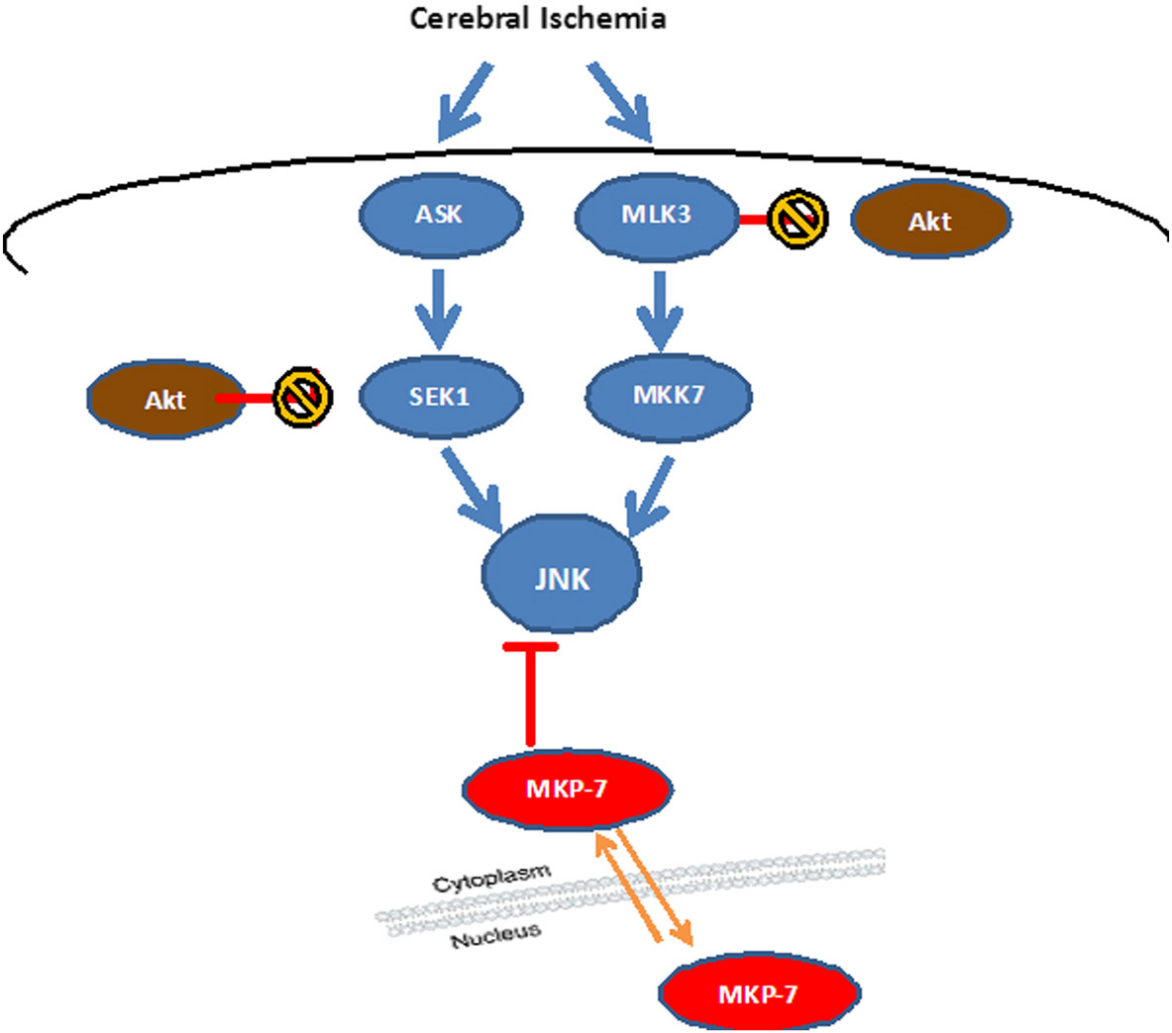
**Model of JNK inhibition after cerebral ischemia/reperfusion.** JNK activity can be elevated by activated ASK-SEK1 or MLK3-MKK-7, or be downregulated by dephosphorylation of JNK at Thr183/Tyr185 by specific phosphatases MKP-7 following ischemia/reperfusion. MKP-7 function is increased by its nuclear-to-cytoplasmic translocation, which ultimately inhibits JNK activity. Active Akt (which inactivates SEK1 by phosphorylation Ser80 and inhibits MKK-7 by MLK3 phosphorylation at Ser674) does not affect down-regulation of JNK activity induced by MKP-7 after ischemia in the rat hippocampus.

In summary, MKP-7 was demonstrated in this study to play key roles in JNK inactivation during cerebral ischemia, and this inhibition of JNK occurred independently of PI3K/Akt pathway. Shuttling of MKP-7 was shown to be exported from the nucleus to downregulate cytoplasmic JNK activity after ischemia (Figure 7). These findings enrich our understanding of the mechanism of JNK inactivation after ischemia. Thus, exploring drugs which can elevate MKP activity and duration may provide new targets for treatment of ischemia/reperfusion injury.

## Methods

### Surgical procedure

Animal surgery was performed in compliance with the Institutional Animal Care and Use Committee, conforming to international guidelines on the ethical use of animals, and was approved by the Biological Research Ethics Committee of Nanjing Medical University (permit number: NYLL2010-0009). Every effort was made to minimize the number of animals used and their suffering. Adult male Sprague–Dawley rats weighing 250–300 g (obtained from the Experimental Animal Center of Nanjing Medical University) were maintained at room temperature and given free access to food and water. Before surgery, the animals were deprived of food overnight. The rats were anesthetized by intraperitoneal injection of 20% chloral hydrate (250 mg/kg) and surgically prepared for four-vessel occlusion according to the method described by Pulsinelli et al. [33]. Bilateral vertebral arteries were electrocauterized, and both common carotid arteries were dissected free. Twenty-four hours later, both common carotids were occluded with aneurysm clips. Rats were kept awake during the periods of ischemia and subsequent reperfusion, which eliminated the potential effects of sedatives such as chloral hydrate on the results of the experiment. The selected rats matched the following criteria: maintenance of dilated pupils, loss of cornea reflex, completely flat electroencephalogram, rigor of the extremities and vertebral column, and maintenance of rectal temperature at approximately 37°C. Rats in the sham group received the same surgical procedures except for the occlusion of the four arteries.

### Infusion and administration of drugs

The phosphoinositide-3 kinase (PI3K)/Akt inhibitor LY294002 (LY, 10 mM, Calbiochem-Novabiochem Corp., San Diego, CA, USA), MAPK phosphatase or protein synthesis inhibitor cycloheximide (CHX, 177 mM, Sigma-Aldrich, St. Louis, MO, USA), the Dusp6 inhibitor (E)-2-benzylidene-3-(cyclohexylamino)-2,3-dihydro- 1H-inden-1-one (BCI, 28 mM, Sigma-Aldrich Co.) and MKP-7 nuclear export inhibitor Leptomycin B (LMB, 0.2 μg/μl, Enzo Life Sciences, Farmington, NY, USA) or the same volume of vehicle (2 μL) were injected into the cerebral ventricle (0.8 mm posterior and 1.5 mm lateral to the bregma; 3.5 mm deep) using a micro-injector 30 min before ischemia induction according to previous previously published reports [34,35]. The injector was retained in place for another 5 min after the injection, avoiding any possible backflow of liquid along with the injection void.

### *In vivo* MKP-7 siRNA transfer

Five microliters of MKP-7 siRNA (20 μM, RiboBio Co., Guangzhou, China) or control siRNA-A (20 μM, RiboBio) was diluted with the same volume of transfection reagent (Santa Cruz Biotechnology, Santa Cruz, CA, USA). After gently pipetting the solution up and down, the mixture was then incubated for 45 min at room temperature (~25°C) and injected into the cerebral ventricle (0.8 mm posterior and 1.5 mm lateral to the bregma; 3.5 mm deep) using a micro-injector. This injection was repeated four times, every 12 h starting 2 days before ischemia induction [36].

### Tissue sample preparation

Rats were sacrificed by decapitation at various times: 10 min after ischemia and 15 min, 1, 2, 4, 6 and 24 h after reperfusion. The hippocampal tissues were removed on ice and homogenized in 1:10 (W/V) ice-cold homogenization buffer A [50 mM HEPES (pH 7.4), 100 mM KCL, 1 mM Na_3_VO_4_, 50 mM NaF and 1 mM PMSF] containing 1% mammalian proteinase inhibitor cocktail (Sigma-Aldrich). Proteins in the cytoplasm and membrane were extracted after centrifugation at 800 × *g* and 4°C. After this step, the supernatant was centrifuged at 14,000 × *g* and 4°C to harvest cytoplasmic proteins. The resulting pellet after the first centrifugation was resuspended in homogenization buffer B [50 mM HEPES (pH7.4), 100 mM KCL, 1 mM Na_3_VO_4_, 50 mM NaF, 1 mM PMSF, 1 mM DTT, 1% cocktail, and 5% SDS] and kept on ice for 30 min, followed by ultrasonic disruption for 6 s, repeated 6 times and then centrifugation at 14,000 × *g* and 4°C. The supernatants containing the nuclear or cytoplasmic proteins were extracted and then stored at −80°C until assayed. The protein concentrations of the extracts were determined according to the Bradford method using bovine serum albumin (BSA) as a standard [37].

### Western blot

Denatured samples were separated by 10% SDS-PAGE and electrotransferred onto nitrocellulose membranes (NC, pore size, 0.2 μm). The proteins on the membrane were probed with primary antibodies against JNK, phosphorylated JNK at Thr183/Tyr185, Akt, phosphorylated Akt at Ser473, phosphorylated SEK1 at Ser80, phosphorylated MKK7 at Ser271/Thr275, MKP-7 (Santa Cruz Biotechnology) and β-actin (Boster Biotechnology, WuHan, HB, China) at 4°C overnight. Following three 5–10 min washes in Tris-buffered saline, 0.1% Tween-20 (TBST), the immune complexes on the membrane were then incubation with horseradish peroxidase (HRP)-conjugated anti-rabbit or mouse secondary antibody (1:5000, ZhongShan Golden Bridge Biotechnology, Peking, China) for 2 h. Following the incubation, the membranes were then washed in TBST three times for 10 min each time. The bands on the membranes were scanned and analyzed using an image analyzer.

### Phosphatase activity assay

The primary rabbit antibody to MKP-7 (1–2 μg) was added to each sample of total proteins (200 μl). After shaking overnight at 4°C, protein A/G PLUS-Agarose (10 μl, Santa Cruz Biotechnology) was added and incubated with shaking for 2 h at 4°C. The protein A/G PLUS-Agarose was precipitated by centrifugation at 5000 × *g* and washed three times with immunoprecipitation buffer. The washed agarose was resuspended in 200 μl phosphatase assay buffer containing 20 mM pNPP and 50 mM imidazole (pH 7.5) (both from Enzo Life Sciences, Farmington, NY, USA) and incubated for 2 h at 30°C. The reaction was stopped by the addition of 800 μl of 0.25 N NaOH, and p-NPP hydrolysis was measured by absorbance at 410 nm.

### Data and statistical analysis

Data were documented as means ± SD from at least three independent rats. One-way analysis of variance (ANOVA) followed by Duncan’s new multiple range method was applied to analyze the statistical results. *P*-values < 0.05 were considered significant.

## Competing interests

The authors declare that they have no competing interest.

## Authors’ contributions

JZ, WS, SS carried out the 4-VO model, sample preparation and Western blot analysis. JZ participated in MKP7 activity assay. JZ, HG participated in the I.c.v. infusion. JZ, WS, LG drafted the manuscript. HW, JG conceived of the study, and participated in its design and coordination. All authors read and approved the final manuscript.
